# COVID-19: some unanswered questions

**DOI:** 10.1136/postgradmedj-2020-137951

**Published:** 2020-05-13

**Authors:** Philip D Welsby

**Affiliations:** Retired, Edinburgh, UK

**Keywords:** epidemiology, health services administration & management, organisation of health services, infectious diseases, public health

In 2003, severe acute respiratory syndrome (SARS) spread through 26 countries, infecting at least 8098 and causing at least 774 deaths (a case fatality rate of 9.6%). Middle East respiratory syndrome (MERS) by January 2020 caused 2519 cases and 866 deaths (a case fatality rate of 34%). SARS and MERS are coronaviruses and both are not as easily transmitted as COVID-19 because they require close contact with those infected (or also with camels in the case of MERS), and infected humans tend not to transmit before they have symptoms. Transmission of both mostly occurred within healthcare settings and could be controlled by improving infection control in hospitals.

In 2015, Bill Gates in a TED lecture warned that we were more at risk of a global pandemic (he thought it would be influenza) than we were from nuclear war.

COVID-19 probably first entered the human population in China in November 2019 in Wuhan and was first identified as such in December 2019. It spreads easily with a R_0_ (basic reproduction number) that represents the average number of people the average infected person would infect being between 1.5 and 3.5, depending on the surrounding circumstances. While a large proportion of infections are asymptomatic, there is a significant mortality rate (about 3.4% worldwide). Survival rates are worse in the elderly, in men and in those with comorbidities. There are no suitable mammal models to study.

Because there is a significant proportion of asymptomatic infectious people, monitoring of epidemics necessitates screening to determine (1) the proportion of the population that is actively infected and or (2) the total number of those who have been infected. Both require screening. To gain significant data, then whole populations or representative samples have to be tested. In many circumstances, only those with high probability are tested.

*DNA polymerase techniques* on throat swabs (notably real-time reverse transcription PCR) can identify the actively infected, but such tests will need to be repeated, especially in healthcare staff who are both at increased risk of infection and could provide an increased risk of infection to their contacts.

*Antibody tests* in theory can reveal who has been infected. However, such tests may not provide 100% reliable results, including the fact that their sensitivity will vary according to how common the infection is. If an infection is common, then a very sensitive test will identify all those infected and also a small number of false positives, but when the infection becomes less common, then the proportion of false positives will rise and a positive test could become less useful. Moreover, for how long would the antibody-person be immune?

*Counting the number of hospital deaths* attributed to COVID-19 may be a guide to an epidemic, but deaths may be difficult to count in the community. In any case, changes in death numbers usually lag a few weeks behind the time of infection.

Would a lower infecting dose cause the following illness to be less severe? Does the virus need several extra doubling times to exert its effects such that in this gained time host responses will be in a better position to combat the infection in high-risk groups or in groups where medical care is minimal? Could low-dose vaccination with COVID-19 itself be useful? Shakespeare’s *Hamlet* (not an epidemiologist) suggested, ‘Diseases desperate grown, By desperate appliance are relieved, Or not at all’.

All the aforementioned are key questions, the answers to many of which are not known at the time of writing and, even if they were, the answers might change with the passage of time.

## Various countries have made various policy choices

At the time of writing (April 2020), COVID-19 has probably been in the human population for only about 6 months. In most countries, there are concerns about how the epidemic was initially handled, and it is possible to predict some damming retrospective judgements. However, we should concentrate on where we are, not where we might have been. Recriminations should wait.

Many important decisions have to be made based on incomplete information. Most COVID-19 decisions have to be made on speculations (guesswork and wishful thinking), on hypotheses (propositions made as a basis for reasoning, without an assumption of its truth) or on theories (suppositions or systems of ideas explaining something based on general principles). All COVID-19 decisions have to be made at the time ‘We have to start from where we are’ guided by the experiences of other countries that are ahead of us in the epidemic.

Pandemics usually reveal inequalities and the poor, or those in unstable employment or in crowded accommodation, or with underlying health issues, or where healthcare is less affordable, or are in the less well educated will suffer the most. They will also comply less with restrictions. Ideologies, power blocks, leaders, social cohesion beliefs, the relevance of centralised or regional decision making, the abilities of popularism (political doctrines chosen to appeal to a majority of the electorate), welfare states (usually capitalist nations that recognise that food, shelter, education and medicine are basic rights to be ensured by government actions) and authoritarianism are all being stress tested by COVID-19. In the future, it will be interesting to judge how these societal systems played out when confronting the conflicting requirement to reconcile conflicting priorities of health and economic factors that involve conflicts between responding and planning *for* deaths (‘How should we cope with these’) and actually planning deaths. ‘We will have to accept that we will cause deaths whatever policy we adopt’.

There is only one initial response to COVID-19 that reduces infection rates and death rates. Dramatic quarantine ‘total lockdown’ measures. Some countries, including China, South Korea, Hong Kong, Taiwan and Singapore, hit the epidemic hard and early with lockdown quarantine to reduce the epidemic. Such countries perhaps tend towards acceptance of authoritarianism and their citizens less rebellious than in other countries. New Zealand did similarly. I could not possibly comment on the US responses. However, on what criteria and at what speed should liberalisation of quarantine measure occur to avoid re-emergences?

There are in theory three final paths out of the COVID-19 crisis:

*First*, a vaccine. Even a perfect vaccine would be difficult to evaluate with changing risks in the community. How protective would a vaccine be and for how long would it be effective?

*Second*, the identification of a treatment, either preventative or curative, so that the disease becomes a considerably less worrisome prospect even for those with comorbidities.

*Third*, herd immunity, when enough of the population has acquired and survived COVID-19 and thus developed immunity with the infection persisting at a low level. Currently the only, not entirely definitive, way of estimating this is by measuring antibodies such that there would not be enough opportunities for disease transmission for the virus to continue circulating through populations with an R_o_ of less than 1, but the risk would not disappear entirely. Moreover, how should immunity be monitored if antibody testing may not reflect herd immunity? Allowing herd immunity to develop initially would result in a huge spike in hospitalisations and deaths that could overwhelm most healthcare services, and that is why flattening such spikes by quarantine was indicated. With flattening, there would still be illness and deaths but at a controlled slower rate and hopefully also smaller numbers, such that healthcare services could cope.

There is a lot of opinion and numerous contributions by official and unofficial organisations and individuals who think their “single issue advice” should be followed. No one individual has the expertise required for management of all the complexities. Committees are required, including microbiologists, infectious diseases doctors, public health doctors, epidemiologists, hospital and general practice representatives, epidemic mathematical modellers and economic advisers. Politicians have the responsibility to deliver decisions when, especially when, information is imperfect. How many people would be infected if we did nothing? What would the epidemic curve look like in various situations? What proportion of those infected would infect others in various situations? How many of which population groups would require what extra healthcare services in various situations? What would be the effect of various measures at various times? What economic impacts might there be when these in themselves affect mortality rates?

I predict that COVID-19 will cause two significant changes in political thought. First, it has to be realised that globalisation of such epidemics, and there will be more to come, will demand an integrated globalised response. Second, in 1987, Margaret Thatcher, the UK Prime Minister, said that ‘There is no such thing as society… the quality of our lives will depend on how much each of us is prepared to take responsibility for ourselves and each of us prepared to turn round and help by our own efforts those who are unfortunate’. The current UK Prime Minister in March 2020 presented a new synthesis, ‘There really *is* such a thing as society’.

Finally, it is important to realise that everyone, no matter where they are, for better or worse, has to rely on their existing rulers or governments.

